# A Prospective Study of Lupus and Rheumatoid Arthritis in Relation to Deployment in Support of Iraq and Afghanistan: The Millennium Cohort Study

**DOI:** 10.4061/2011/741267

**Published:** 2011-11-14

**Authors:** Kelly A. Jones, Nisara S. Granado, Besa Smith, Donald J. Slymen, Margaret A. K. Ryan, Edward J. Boyko, Gary D. Gackstetter, Christopher J. Phillips, Tyler C. Smith

**Affiliations:** ^1^Deployment Health Research Department, Naval Health Research Center, 140 Sylvester Road, San Diego, CA 92106-3521, USA; ^2^Graduate School of Public Health, San Diego State University, San Diego, CA 92182-4162, USA; ^3^Occupational Health Department, Naval Hospital Camp Pendleton, Camp Pendleton, CA 92055, USA; ^4^Department of Veterans Affairs Puget Sound Health Care System, Seattle Epidemiologic Research and Information Center, Seattle, WA 98108-1532, USA; ^5^Analytic Services, Inc. (ANSER), Arlington, VA 22206-2233, USA

## Abstract

The objective of this study was to prospectively assess the association between deployment in support of the operations in Iraq and Afghanistan and newly reported lupus and rheumatoid arthritis while also considering the effects of demographic, behavioral, and occupational characteristics. A total of 77,047 (2001–2003) and 31,110 (2004–2006) participants completed the baseline Millennium Cohort questionnaire and were resurveyed approximately every 3 years. Longitudinal analyses were used to assess the adjusted association between deployment to Iraq and Afghanistan with and without combat exposures and newly reported disease. After adjusting, deployment was not significantly associated with newly reported lupus compared with nondeployers. However, compared with nondeployers, deployers with and without combat exposures were significantly less likely to newly report rheumatoid arthritis. Women, non-Hispanic black, and Hispanic participants had a significantly elevated risk for both diseases. Overall, deployment was not associated with an increased risk of newly reported lupus or rheumatoid arthritis.

## 1. Introduction

Representing a diverse family of chronic disease, autoimmune diseases are disabling and have a variety of natural histories. It is estimated that rheumatoid arthritis (RA) and systemic lupus erythematosus (SLE) affect considerable numbers of US residents (460 per 100,000 persons and 40–150 per 100,000, resp.) [1–5]. The etiologies of these conditions are unknown, and both diseases cause chronic, systemic inflammation resulting in tissue and/or organ damage [6]. Several studies document how these diseases adversely affect the general health of many in the US population [2–5]; however, research is sparse in the US military population on not only the frequency but also the risk factors associated with their occurrence.

Military personnel deployed to combat regions during the current operations in Iraq and Afghanistan are often exposed to stressful situations putting this population at higher risk for stress-related medical conditions and lifestyle choices associated with poorer health outcomes [7–11]. There have been concerns with previous military operations that service members potentially have a higher risk of SLE and RA due to traumatic stressors experienced during deployment [12–14]. Furthermore, military personnel may experience several environmental and occupational exposures, including pesticides (environmental and topical), paints, solvents, and silica dust that have been associated with SLE and RA in the general US population [1, 15–21]. It is important to understand potential factors that may increase the risk of these complex diseases, especially when military deployments may include unique environmental and occupational exposures [10, 22]. Using data from the Millennium Cohort Study, these medical outcomes were prospectively investigated in relation to demographic, occupational, and behavioral characteristics.

## 2. Materials and Methods

### 2.1. Study Population

Participants were from the Millennium Cohort Study, a prospective, population-based sample consisting of a weighted random sample of all US service members on rosters as of October 2000 and October 2003, who were enrolled beginning in 2001 and 2004, respectively. The Cohort was primarily designed to evaluate long-term health effects related to military service over a 21-year time period. This study includes participants from the 2001 and 2004 enrollment cycles. A more detailed description of the Millennium Cohort Study has been published elsewhere [23–25].

For the 2001 enrollment, a total of 77,047 participants voluntarily consented and completed the baseline questionnaire (2001–2003), with 63,372 completing at least one of the follow-up questionnaires (2004–2006, 2007-2008). For the 2004 enrollment, 31,110 consented and completed the baseline questionnaire (2004–2006) with 17,152 completing the first follow-up questionnaire (2007-2008). Of the 80,524 participants who completed the baseline and follow-up questionnaires, exclusions included missing or affirmative self-report of lupus or RA at baseline, missing responses to questions relating to lupus or RA on at least one follow-up questionnaire, self-reported disease at first follow-up but not at the second follow-up, self-reported disease prior to first deployment, and affirmatively responded to all provider-diagnosed disorders. This left a total population of 76,425 and 74,643 participants for the analyses of lupus and RA, respectively.

### 2.2. Data Collection

Fixed and time-varying demographic and military characteristics were obtained from the electronic personnel files maintained at the Defense Manpower Data Center and linked to each participant at baseline and each follow-up. Data included sex, birth year, race/ethnicity, education, marital status, branch of service, service component, military pay grade, military occupation, and deployment experience in support of the operations in Iraq and Afghanistan from 2001 to 2008.

Participants were defined as deployers at baseline if they had deployed for 1 or more days in support of operations in Iraq and Afghanistan prior to baseline and were defined as deployers at each follow-up if they were deployed for 1 or more days between follow-up survey assessments. Deployers were further classified as with or without combat experiences on each follow-up based on a positive self-report to personally witnessing or being exposed in the past 3 years to a person's death due to war or disaster, physical abuse, dead or decomposing bodies, maimed soldiers or civilians, or prisoners of war or refugees. Combat experiences were not assessed at baseline due to uncertainty of deployment-exposure relation because the question was addressed in the context of “ever” rather than the “past 3 years.” Participants not meeting the deployment definition were considered nondeployers.

Using baseline and follow-up surveys, time-varying general health, behavioral, and occupational characteristics were included to evaluate whether certain subpopulations were more likely to newly report lupus or RA. General health was investigated using the physical and mental component summary scores from the Medical Outcomes Study Short Form 36-Item Health Survey for Veterans [26–31]. Participants' component scores were categorized into quartiles. Body mass index was classified as normal/underweight (<25 kg/m^2^), overweight (25 to <30 kg/m^2^), and obese (≥30 kg/m^2^). Nonsmokers, past smokers, and current smokers were identified using survey questions addressing lifetime smoking of at least 100 cigarettes (5 packs), a successful attempt to quit smoking, and cigarette use in the past year. Alcohol misuse was evaluated using the CAGE questionnaire (cutting down, annoyance by criticism, guilty feeling, and eye openers) [32, 33]. Finally, environmental and occupational exposures were derived from an affirmative self-report of ever being exposed prior to newly reporting disease to the following: chemical and biological warfare agents, occupational hazards requiring personal protective equipment; routine skin contact with paint, solvents, or substances, microwaves (excluding microwave ovens), and topical (creams, sprays, uniform treatments) and environmental pesticides.

### 2.3. Outcomes

Lupus and RA were investigated using the baseline survey question “Has your doctor or other health professional ever told you that you have any of the following conditions?” At follow-up, participants were asked the same question, but in the context of “in the last 3 years.” Newly reported lupus and RA were defined as an affirmative self-report to these diseases on either of the follow-up surveys among participants reporting no prior condition. Those who did not report an outcome of interest constituted the nondiseased control comparison groups.

### 2.4. Statistical Analyses

Descriptive and univariate analyses were conducted to examine unadjusted associations of the study outcomes with demographic, occupational, and behavioral risk factors. Separate models were developed for both newly reported lupus and RA. Longitudinal analyses were performed using the generalized estimating equations method to compare the adjusted association between deployment status and both outcomes [34, 35]. All analyses adjusted for time calculated as the number of years between baseline and each follow-up survey. A model analysis was performed using a variation inflation factor greater than four to indicate a potential problem with multicollinearity. Confounders were defined as variables changing the association between each disease outcome and deployment status by more than 10% [36]. Variables that were not confounders nor statistically significant in the model at *P* < 0.05 were manually removed using a backward reduction method to establish the final models. If a variable was neither confounding nor significant but was consistently associated with either outcome in previous published literature, the variable remained in the model. Statistical analyses were performed using SAS software version 9.2 (SAS Institute, Inc., Cary, NC, USA).

## 3. Results

### 3.1. Lupus

Baseline characteristics and deployment status stratified by whether participants reported lupus at follow-up are shown in Table 1. Over an average of 5.6 years between baseline and last follow-up, the cumulative incidence of lupus was 0.34 per 1000 person-years. Lupus was newly reported among 0.2% of nondeployers and 0.1% of deployers. Deployers were proportionately more likely to be male, younger, less educated, and in the Army compared with nondeployers.

The results from the univariate analysis and the final adjusted model are shown in Table 2. Subgroups proportionately more likely to newly report lupus were female, non-Hispanic black and Hispanic, not married, and of lower mental and physical health. The following variables were removed from the full adjusted model (not shown), because they were not significant (*P* > 0.05) and did not confound the results: education, marital status, service component, pay grade, service branch, occupation, body mass index, smoking status, alcohol misuse and exposure to chemical and biological warfare agents, occupational hazards requiring personal protective equipment, skin contact with paints, solvents, or substances, microwaves (excluding microwave ovens), and topical and environmental pesticides. The final adjusted model revealed deployments with and without combat exposures were not significantly associated with newly reported lupus when compared with nondeployers (*P* = 0.37). Women were nearly twice as likely (adjusted odds ratio (AOR) = 1.81, 95% confidence interval [CI]: 1.20–2.72) to newly report lupus compared with men. Non-Hispanic black (AOR = 2.66, 95% CI: 1.70–4.17) and Hispanic (AOR = 2.35, 95% CI: 1.27–4.36) participants had over two times the risk of newly reported lupus than non-Hispanic white participants. Those reporting low mental and physical health were at significantly higher risk of newly reported lupus when compared with those reporting high mental or physical health (>75th percentile).

### 3.2. Rheumatoid Arthritis

Baseline characteristics and deployment status stratified by whether participants reported RA at follow-up are shown in Table 1. The cumulative incidence of RA between 2001 and 2008 was 3.30 per 1000 person-years. RA was newly reported among 1.9% of nondeployers and 1.1% of deployers. Similar to lupus deployers were proportionately more likely to be male, younger, less educated, and in the Army compared with nondeployers.

Results for the univariate analysis and the final adjusted model are shown in Table 3. Subgroups proportionately more likely to newly report RA were female, non-Hispanic black and Hispanic, less educated, overweight, past and current smokers, alcohol misusers, of lower mental and physical health, having had unknown exposure to occupational hazards requiring personal protective equipment, and having had exposure to skin contact with paints, solvents, or substances; microwaves; and environmental or topical pesticides. To establish the final model, the following covariates were removed from the full model (not shown), because they were not significant (*P* > 0.05) and were not determined to be confounders: marital status, alcohol misuse, and exposure to occupational hazards requiring personal protective equipment, routine skin contact with paints, solvents, or substances, and environment and topical pesticides. The final adjusted model revealed those deployed with (AOR = 0.74, 95% CI: 0.62–0.88) and without (AOR = 0.59, 95% CI: 0.48–0.73) combat exposures were significantly less likely to newly report RA. Those significantly less likely to newly report RA were younger; Reserve or National Guard; officers; Air Force, Navy, or Coast Guard; and health care specialists. Women were 1.26 (95% CI: 1.08–1.47) times more likely to report RA than men. Non-Hispanic black (AOR = 1.77, 95% CI: 1.51–2.08) and Hispanic (AOR = 1.40, 95% CI: 1.11–1.76) participants had a significantly higher risk of disease compared with non-Hispanic white participants. Those with some college, obese respondents, and current and past smokers also had a significantly elevated risk of RA. Lower mental and physical health, exposure to chemical and biological warfare agents, and “do not know” response to exposure to microwaves were significantly associated with an increased risk of newly reported RA.

## 4. Discussion

Concern was raised after the 1991 Gulf War [13, 14] of a possible association between service in this conflict and a higher risk of SLE and RA. This potential association continues to be of interest in light of stressful military deployments currently in Iraq and Afghanistan [7–9, 11, 25, 37, 38]. This is the first study to prospectively document, in a large population-based military cohort, newly reported lupus and RA in the context of deployment while differentiating stresses of combat experiences. Compared with nondeployers, deployment with and without combat exposures was not found to be a significant risk factor for newly reported lupus. However, deployment with and without combat exposures was significantly associated with a decreased risk of newly reported RA compared with nondeployers. To better assess the full effects associated with stressful exposures during deployment, deployers with combat exposures were compared with deployers without combat exposures. Compared with noncombat deployers, those with combat exposures had an increased risk of newly reported RA though not significant (AOR = 1.24, 95% CI: 0.96–1.61). This may indicate a stress-induced pathway that elevates the risk for RA among deployers who experience combat [12, 39, 40]. Though long-term follow-up of the Cohort will yield further insight into deployment-related effects, these results are reassuring that compared with nondeployers, there is no apparent increase in risk for newly reported lupus or RA in relation to deployment in support of Iraq and Afghanistan.

The cumulative incidence of lupus reported in this study was elevated compared with previous reports for the US population (systemic lupus erythematosus: 0.02 to 0.08 per 1000 person-years) [41–43]. It is difficult to compare these rates, because military personnel may have been exposed to unique environmental and occupational risk factors not typically experienced in the general population. However, misclassification of this disorder may have occurred, because participants reporting lupus may have been diagnosed with any form of lupus and not SLE specifically. This is due to the questionnaire not restricting which type of lupus to be reported. 

Previous literature has documented that environmental and occupational exposures were associated with an increased risk of lupus [16, 18–20]; however, these predictors were not strong risk factors for lupus development in this study and were not included in the final model. Though incidence rates have been reported to be much higher for women, and non-Hispanic black and Hispanic participants in the US population [3, 41, 43, 44], the incidence of lupus has not been thoroughly investigated in a military population, making an appropriate comparison difficult. Despite these differences, lower mental and physical health was associated with increased risk of lupus, which was consistent with previous research in the US population [45]. 

Similar to lupus, there was a higher cumulative incidence of self-reported RA in this study compared with previous results in the US population (0.24 to 0.68 per 1000 person-years) [1, 5, 42]. This may be a result of those with any form of arthritis endorsing RA, as the questionnaire did not provide a definition of RA. Also, the incidence rates for women and non-Hispanic black and Hispanic participants have been reported higher for the US population than in the current study [1, 5]. These findings, however, were consistent with previous reports of greater RA risk associated with lower mental and physical health status [45, 46], higher body mass index [1, 47, 48], smoking [1, 47, 48], and less education [1, 47]. Additionally, the documented average age of new-onset RA is between 30 and 55 years of age among the general population [1], and the current study consistently found a lower risk associated with younger age. 

To our knowledge, an increased risk of RA among those self-reporting exposure to chemical or biological warfare agents has not been reported. Due to the lack of evidence of chemical or biological warfare agents existing in the current operations in Iraq and Afghanistan, this association may be a self-perceived exposure or the result of overreporting bias. In addition, the association between RA and exposure to microwaves (excluding microwave ovens) has not been documented. While in vivo and in vitro studies suggest low exposures to microwaves may cause changes in the immune system, and neurological and behavioral effects, the true biological responses due to microwave exposures are still under investigation [49].

This study had several limitations. The current analyses used self-reported data from the Millennium Cohort questionnaire; however, previous studies of this Cohort suggest it is representative of US military personnel, the data reported by its participants are reliable and were unaffected by the participant's health status prior to enrollment [22, 23, 36, 50–57]. Also, the questionnaire did not specify which type of lupus or define RA which may have resulted in misclassification of both outcomes. With a large sample size and population-based design, conducting clinical examinations to confirm self-reported diagnoses was not feasible. The documented validity of self-reported provider-diagnosed data varies widely for lupus (21%–84%) and RA (7%–96%) [58–63]. Therefore, an electronic medical records review was performed for both outcomes using ICD-9 codes in Armed Forces Health Longitudinal Technology Application and Military Health System databases. This review could only be conducted for active-duty members diagnosed while in service. Confirmation of 13 out of 33 (39.4%) lupus and 39 out of 281 (13.9%) RA self-reported diagnoses were found. Thus, there may be substantial misclassification of both outcomes. The sensitivity analyses among the confirmed diagnoses resulted in stability of the measures of association between deployment and lupus and RA (see “Confirmed case models”, Tables 2 and 3). 

Another important limitation was lupus and RA are rare outcomes resulting in few newly reported cases occurring during the average 5.6 years of follow-up. This may ultimately affect the precision of the estimates that will only be lessened with additional follow-up and subsequent cases. Also, deployment included those deployed for 1 or more days, so those on shorter deployments had fewer days exposed than those on longer deployments. Furthermore, deployed participants that did not affirmatively respond to the questions defining combat exposure may have been exposed to combat situations that were not addressed in this study including receiving small arms fire, or being attacked or ambushed. Lastly, the Millennium Cohort Study does not obtain information on diet, family history, genetic risk factors, or medication, making it difficult to thoroughly examine these exposures as possible risk factors for these diseases.

Despite these limitations, this study has several strengths. This is the first study to prospectively investigate whether military deployment status is associated with newly reported provider-diagnosis of lupus and RA, while also being able to evaluate specific military exposures and adjust for negative health behaviors such as alcohol misuse and tobacco use. Additionally, the prospective design of the Cohort allowed for assessment at baseline and follow-up of the same individuals accounting for time-varying covariates. Lastly, a large sample size, representing both men and women, all branches of the military, active duty, and Reserve and National Guard personnel during and after service, enhanced statistical power for assessing these chronic diseases while considering multiple potential confounders.

In conclusion, newly reported lupus was not associated with military deployment in support of the current operations in Iraq and Afghanistan when compared with nondeployers. These findings, however, suggest a significantly decreased risk of newly reported RA for deployers with and without combat exposures and may be due to a selection effect for deployment. Demographic and general health characteristics and specific exposures were also associated with lupus and RA development in this population. While this study did not find deployment to be a significant risk factor for development of lupus or RA, it is important to further investigate these associations in the future, as the incidence of lupus and RA may progress over time. This study highlights the strengths of prospectively addressing long-term health concerns associated with occupational and environmental exposures among military personnel.

## Figures and Tables

**Table 1:** Baseline* characteristics of Millennium Cohort participants with newly reported lupus and rheumatoid arthritis (2001–2008).

Characteristics	Lupus				Rheumatoid arthritis			
	Disease		No disease		Disease		No disease	
	*n* = 115		*n* = 72,576		*n* = 1,100		*n* = 69,924	
	*n*	%^†^	*n*	%^†^	*n*	%^†^	*n*	%^†^
Deployment^‡^								
Nondeployed	75	65.2	39,874	54.9	744	67.6	38,111	54.5
Deployed without combat	22	19.1	17,985	24.7	174	15.8	17,594	25.2
Deployed with combat^§^	18	15.7	14,717	20.3	182	16.6	14,219	20.3
Sex								
Male	56	48.7	51,240	70.6	760	69.1	49,321	70.5
Female	59	51.3	21,336	29.4	340	30.9	20,603	29.5
Birth year								
Before 1960	22	19.1	13,686	18.9	381	34.6	12,629	18.1
1960–1969	41	35.7	24,131	33.3	470	42.7	22,996	32.9
1970–1979	41	35.7	24,020	33.1	197	17.9	23,645	33.8
1980 and later	11	9.6	10,739	14.8	52	4.7	10,654	15.2
Race/ethnicity								
Non-Hispanic white	58	50.4	51,610	71.1	673	61.2	49,882	71.3
Non-Hispanic black	32	27.8	8,723	12.0	244	22.2	8,214	11.8
Hispanic	13	11.3	4,883	6.7	90	8.2	4,718	6.8
Other	12	10.4	7,360	10.1	93	8.5	7,110	10.2
Education								
High school or less	67	58.3	37,153	51.2	572	52.0	35,815	51.2
Some college	25	21.7	15,797	21.8	297	27.0	15,061	21.5
Bachelor's or higher degree	23	20.0	19,626	27.0	231	21.0	19,048	27.2
Marital status								
Married	66	57.4	42,522	58.6	793	72.1	40,514	57.9
Not married	49	42.6	30,054	41.4	307	27.9	29,410	42.1
Service component								
Active duty	64	55.7	40,745	56.1	640	58.2	39,235	56.1
Reserve/Guard	51	44.4	31,831	43.9	460	41.8	30,689	43.9
Military pay grade								
Enlisted	97	84.4	55,262	76.1	935	85.0	53,068	75.9
Officer	18	15.7	17,314	23.9	165	15.0	16,856	24.1
Service branch								
Army	63	54.8	33,856	46.7	619	56.3	32,349	46.3
Air Force	30	26.1	21,557	29.7	272	24.7	20,924	29.9
Marine Corps	4	3.5	3,880	5.4	43	3.9	3,765	5.4
Navy/Coast Guard	18	15.7	13,283	18.3	166	15.1	12,886	18.4
Occupation								
Combat specialist	16	13.9	14,058	19.4	185	16.8	13,588	19.4
Health care specialist	23	20.0	8,259	11.4	90	8.2	8,054	11.5
Functional support	33	28.7	14,085	19.4	291	26.5	13,428	19.2
Electrical/mechanical	12	10.4	10,156	14.0	155	14.1	9,744	13.9
Service support	10	8.7	6,433	8.9	125	11.4	6,139	8.8
Other	21	18.3	19,585	27.0	254	23.1	18,971	27.1
Body mass index (kg/m^2^)								
Normal/underweight (<25)	56	48.7	29,520	40.7	320	29.1	28,790	41.2
Overweight (25 to <30)	45	39.1	35,874	49.4	604	54.9	34,441	49.3
Obese (≥30)	14	12.2	7,182	9.9	176	16.0	6,693	9.6
Smoking status								
Nonsmoker	68	59.1	42,572	58.7	552	50.1	41,285	59.0
Past smoker	21	18.3	17,425	24.0	321	29.2	16,613	23.8
Current smoker	26	22.6	12,579	17.3	228	20.7	12,026	17.2
Alcohol misuse^¶^								
No	91	79.1	58,901	81.2	894	81.3	56,766	81.2
Yes	24	20.9	13,675	18.8	206	18.7	13,158	18.8
Mental component score								
>75th percentile	26	22.6	20,760	28.6	299	27.2	19,974	28.6
>50th to 75th percentile	27	23.5	17,759	24.5	248	22.6	17,166	24.6
>25th to 50th percentile	23	20.0	16,266	22.4	182	16.6	15,721	22.5
0 to 25th percentile	39	33.9	17,791	24.5	371	33.7	17,063	24.4
Physical component score								
>75th percentile	20	17.4	20,307	28.0	124	11.3	19,756	28.3
>50th to 75th percentile	26	22.6	18,245	25.1	168	15.3	17,664	25.3
>25th to 50th percentile	22	19.1	17,135	23.6	256	23.3	16,552	23.7
0 to 25th percentile	47	40.9	16,889	23.3	552	50.2	15,952	22.8
Chemical and/or biological warfare agents								
No	106	92.2	68,750	94.7	968	88.0	66,446	95.0
Yes, 1 or more times	9	7.8	3,826	5.3	132	12.0	3,478	5.0
Hazards requiring personal protective equipment								
No	65	56.5	31,622	43.6	476	43.3	30,558	43.7
Yes	46	40.0	39,420	54.3	584	53.1	37,921	54.2
Do not know	4	3.5	1,534	2.1	40	3.6	1,445	2.1
Routine skin contact with paint, solvents, substances								
No	74	64.4	50,431	69.5	669	60.8	48,912	70.0
Yes	33	28.7	19,276	26.6	363	33.0	2,719	3.9
Do not know	8	7.0	2,869	4.0	68	6.2	2,719	3.9
Microwaves (excluding microwave ovens)								
No	83	72.2	53,942	74.3	718	65.3	52,253	74.7
Yes	24	20.9	12,818	17.7	232	21.1	12,165	17.4
Do not know	8	7.0	5,816	8.0	150	13.6	5,506	7.9
Pesticides (creams, sprays, uniform treatments)								
No	71	61.7	46,506	64.1	606	55.1	45,091	64.5
Yes	35	30.4	19,652	27.1	378	34.4	18,709	26.8
Do not know	9	7.8	6,418	8.8	116	10.6	6,124	8.8
Pesticides (environment, living facilities)								
No	63	54.8	42.194	58.1	523	47.6	40,985	58.6
Yes	37	32.2	20,508	28.3	410	37.3	19,462	27.8
Do not know	15	13.0	9,874	13.6	167	15.2	9,477	13.6

*Population excludes participants with missing baseline covariates, may not represent whole study population.

^†^Percentages may not sum to 100 because of rounding.

^‡^Deployed in support of the operations in Iraq and Afghanistan.

^§^Self-report of personally witnessing or being exposed to a person's death due to war or disaster, physical abuse, dead and/or decomposing bodies, maimed soldiers or civilians, prisoners of war, or refugees.

^¶^Alcohol misuse is defined as at least 1 positive response to the CAGE questions (i.e., Cutting down, Annoyance by criticism, Guilty feeling, and Eye-openers) [32, 33].

**Table 2:** Odds of newly reported lupus and military deployment, the Millennium Cohort Study (*N* = 77,811).

Characteristics	Unadjusted		Final model*		Confirmed case model^†^	
	OR	95% CI	AOR^‡^	95% CI	AOR^‡^	95% CI
Deployment^§^						
Nondeployed	1.00^¶^		1.00^¶^		1.00^¶^	
Deployed without combat	0.85	0.53–1.35	0.91	0.55–1.50	0.28	0.04–1.82
Deployed with combat^#^	0.64	0.37–1.09	0.66	0.37–1.19	0.32	0.05–2.11
Sex						
Male	1.00^¶^		1.00^¶^		1.00^¶^	
Female	2.42	1.69–3.48	1.81	1.20–2.72	4.24	1.14–15.81
Birth year						
Before 1960	1.00^¶^		1.00^¶^		1.00^¶^	
1960–1969	1.23	0.74–2.04	1.26	0.75–2.11	0.73	0.08–6.67
1970–1979	1.36	0.82–2.27	1.44	0.86–2.44	0.51	0.05–5.22
1980 and later	1.23	0.60–2.51	1.13	0.54–2.37	1.22	0.10–15.21
Race/ethnicity						
Non-Hispanic white	1.00^¶^		1.00^¶^		1.00^¶^	
Non-Hispanic black	3.24	2.12–4.95	2.66	1.70–4.17	2.41	0.60–9.71
Hispanic	2.47	1.35–4.54	2.35	1.27–4.36	1.15	0.13–10.30
Other	1.29	0.69–2.41	1.38	0.74–2.58	0.72	0.09–6.01
Education						
High school or less	1.00^¶^					
Some college	1.41	0.93–2.14				
Bachelor's or higher degree	0.87	0.57–1.31				
Marital status						
Married	1.00^¶^					
Not married	1.67	1.18–2.35				
Service component						
Active duty	1.00^¶^					
Reserve/Guard	0.85	0.60–1.21				
Military pay grade						
Enlisted	1.00^¶^					
Officer	0.58	0.37–0.91				
Service branch						
Army	1.00^¶^					
Air Force	0.69	0.47–1.06				
Marine Corps	0.80	0.32–2.00				
Navy/Coast Guard	0.77	0.47–1.28				
Occupation						
Functional support	1.00^¶^					
Combat specialist	0.53	0.28–1.00				
Health care specialist	1.25	0.73–2.13				
Electrical/mechanical	0.73	0.40–1.35				
Service support	0.71	0.37–1.35				
Other	0.67	0.40–1.11				
Body mass index (kg/m^2^)						
Normal/underweight (<25)	1.00^¶^					
Overweight (25 to <30)	0.82	0.56–1.20				
Obese (≥30)	1.44	0.93–2.24				
Smoking status						
Nonsmoker	1.00^¶^					
Past smoker	1.04	0.69–1.57				
Current smoker	1.27	0.77–2.09				
Alcohol misuse**						
No	1.00^¶^					
Yes	1.13	0.75–1.71				
Mental component score						
>75th percentile	1.00^¶^		1.00^¶^			
>50th to 75th percentile	1.16	0.72–1.85	1.25	0.75–2.07		
>25th to 50th percentile	0.84	0.50–1.42	0.89	0.52–1.53		
0 to 25th percentile	1.96	1.26–3.05	1.68	1.06–2.66		
Physical component score						
>75th percentile	1.00^¶^		1.00^¶^			
>50th to 75th percentile	0.97	0.51–1.84	1.08	0.55–2.14		
>25th to 50th percentile	1.73	0.95–3.14	1.79	0.96–3.34		
0 to 25th percentile	4.27	2.50–7.29	4.34	2.51–7.49		
Chemical/biological warfare agents						
No	1.00^¶^					
Yes, 1 more times	0.68	0.45–1.02				
Hazards requiring personal protective equipment						
No	1.00^¶^					
Yes	0.70	0.49–1.01				
Do not know	1.21	0.43–3.38				
Routine skin contact with paint, solvents, substances						
No	1.00^¶^					
Yes	1.18	0.81–1.73				
Do not know	2.20	1.12–4.32				
Microwaves (excluding microwave ovens)						
No	1.00^¶^					
Yes	1.07	0.69–1.67				
Do not know	1.40	0.82–2.42				
Pesticides (creams, sprays, uniform treatments)						
No	1.00^¶^					
Yes	1.17	0.81–1.69				
Do not know	0.87	0.44–1.73				
Pesticides (environment, living facilities)						
No	1.00^¶^					
Yes	1.15	0.79–1.66				
Do not know	1.02	0.58–1.78				
Time (years)	1.42	1.31–1.55	1.38	1.26–1.50	1.42	1.08–1.88

AOR, adjusted odds ratio; CI, confidence interval; OR, odds ratio.

*Final model established by removing nonsignificant, nonconfounding covariates from the full model.

^†^Confirmed cases model is among active-duty, nonseparated participants. There were not enough confirmed cases for mental and physical component scores to be tested in the model.

^‡^Adjusted odds ratios and associated 95% confidence intervals are adjusted for all other variables in the table.

^§^Deployed in support of the operations in Iraq and Afghanistan.

^¶^Indicates reference category.

^#^Self-report of personally witnessing or being exposed to a person's death due to war or disaster, physical abuse, dead and/or decomposing bodies, maimed soldiers or civilians, prisoners of war, or refugees.

**Alcohol misuse is defined as at least 1 positive response to the CAGE questions (i.e., cutting down, annoyance by criticism, guilty feeling, and eye-openers) [32, 33].

**Table 3:** Odds of newly reported rheumatoid arthritis and military deployment, the Millennium Cohort Study (*N* = 76,003).

Characteristics	Unadjusted		Final model*		Confirmed case model^†^	
	OR	95% CI	AOR^‡^	95% CI	AOR^‡^	95% CI
Deployment^§^						
Nondeployed	1.00^¶^		1.00^¶^		1.00^¶^	
Deployed without combat	0.47	0.39–0.56	0.59	0.48–0.73	0.70	0.33–1.49
Deployed with combat^#^	0.69	0.59–0.81	0.74	0.62–0.88	0.83	0.38–1.77
Sex						
Male	1.00^¶^		1.00^¶^		1.00^¶^	
Female	1.16	1.02–1.31	1.26	1.08–1.47	1.96	0.88–4.37
Birth year						
Before 1960	1.00^¶^		1.00^¶^		1.00^¶^	
1960–1969	0.72	0.63–0.82	0.64	0.55–0.74	0.94	0.30–2.95
1970–1979	0.33	0.27–0.39	0.31	0.26–0.38	0.32	0.09–1.20
1980 and later	0.29	0.22–0.39	0.26	0.19–0.35	0.37	0.08–1.78
Race/ethnicity						
Non-Hispanic white	1.00^¶^		1.00^¶^		1.00^¶^	
Non-Hispanic black	2.27	1.97–2.62	1.77	1.51–2.08	0.58	0.21–1.60
Hispanic	1.49	1.20–1.85	1.40	1.11–1.76	1.17	0.41–3.36
Other	0.92	0.74–1.13	1.00	0.79–1.27	1.26	0.42–3.78
Education						
High school or less	1.00^¶^		1.00^¶^		1.00^¶^	
Some college	1.67	1.47–1.91	1.21	1.05–1.39	1.91	0.92–3.94
Bachelor's or higher degree	0.88	0.76–1.01	1.10	0.93–1.30	1.57	0.64–3.88
Marital status						
Married	1.00^¶^					
Not married	0.98	0.87–1.11				
Service component						
Active duty	1.00^¶^		1.00^¶^		1.00^¶^	
Reserve/Guard	0.83	0.74–0.93	0.73	0.64–0.83	0.14	0.02–1.35
Military pay grade						
Enlisted	1.00^¶^		1.00^¶^		1.00^¶^	
Officer	0.45	0.39–0.53	0.60	0.48–0.74	0.17	0.05–0.61
Service branch						
Army	1.00^¶^		1.00^¶^			
Air Force	0.63	0.55–0.73	0.79	0.67–0.92		
Marine Corps	0.71	0.53–0.96	1.01	0.74–1.37		
Navy/Coast Guard	0.67	0.57–0.79	0.74	0.62–0.89		
Occupation						
Functional support	1.00^¶^		1.00^¶^		1.00^¶^	
Combat specialist	0.80	0.66–0.96	1.09	0.90–1.32	0.78	0.26–2.35
Health care specialist	0.60	0.48–0.76	0.66	0.52–0.85	0.50	0.16–1.55
Electrical/mechanical	1.03	0.85–1.24	1.07	0.87–1.36	1.12	0.46–2.74
Service support	1.19	0.97–1.44	1.11	0.91–1.36	1.52	0.56–4.12
Other	0.87	0.74–1.02	1.00	0.85–1.19	0.40	0.16–0.97
Body mass index (kg/m^2^)						
Normal/Underweight (<25)	1.00^¶^		1.00^¶^		1.00^¶^	
Overweight (25 to <30)	1.34	1.16–1.54	1.11	0.95–1.29	0.77	0.38–1.56
Obese (≥30)	2.41	2.07–2.80	1.41	1.20–1.67	1.20	0.49–2.91
Smoking status						
Nonsmoker	1.00^¶^		1.00^¶^		1.00^¶^	
Past smoker	1.39	1.22–1.58	1.13	0.99–1.30	1.63	0.84–3.14
Current smoker	1.54	1.33–1.80	1.19	1.02–1.40	0.56	0.20–1.56
Alcohol misuse**						
No	1.00^¶^					
Yes	1.18	1.04–1.36				
Mental component score						
>75th percentile	1.00^¶^		1.00^¶^		1.00^¶^	
>50th to 75th percentile	0.73	0.62–0.85	0.89	0.75–1.06	0.72	0.35–1.48
>25th to 50th percentile	0.87	0.75–1.02	1.04	0.88–1.23	0.73	0.36–1.49
0 to 25th percentile	1.67	1.46–1.92	1.64	1.42–1.91	0.85	0.42–1.70
Physical component score						
>75th percentile	1.00^¶^		1.00^¶^		1.00^¶^	
>50th to 75th percentile	1.40	1.05–1.86	1.38	1.02–1.88	1.34	0.37–4.83
>25th to 50th percentile	2.99	2.30–3.90	2.72	2.06–3.61	2.56	0.78–8.35
0 to 25th percentile	12.25	9.60–15.63	9.03	6.95–11.74	7.89	2.48–25.10
Chemical/biological warfare agents						
No	1.00^¶^		1.00^¶^		1.00^¶^	
Yes, 1 or more times	0.98	0.85–1.12	1.47	1.22–1.78	1.43	0.49–4.18
Hazards requiring personal protective equipment						
No	1.00^¶^					
Yes	0.96	0.85–1.07				
Do not know	1.59	1.17–2.18				
Routine skin contact with paint, solvents, substances						
No	1.00^¶^					
Yes	1.39	1.24–1.56				
Do not know	1.74	1.38–2.19				
Microwaves (excluding microwave ovens)						
No	1.00^¶^		1.00^¶^		1.00^¶^	
Yes	1.44	1.26–1.64	1.14	0.99–1.31	0.80	0.36–1.78
Do not know	1.63	1.38–1.92	1.25	1.05–1.50	1.58	0.60–4.16
Pesticides (creams, sprays, uniform treatments)						
No	1.00^¶^					
Yes	1.39	1.24–1.57				
Do not know	1.36	1.13–1.64				
Pesticides (environment, living facilities)						
No	1.00^¶^					
Yes	1.47	1.30–1.65				
Do not know	1.22	1.04–1.45				
Time (years)	1.44	1.40–1.48	1.31	1.27–1.35	1.19	0.99–1.44

AOR, adjusted odds ratio; CI, confidence interval; OR, odds ratio.

*Final model established by removing nonsignificant, nonconfounding covariates from the full model.

^†^Confirmed cases model is among active-duty, nonseparated participants. There were not enough confirmed cases for service branch to be tested in the model.

^‡^Adjusted odds ratios and associated 95% confidence intervals are adjusted for all other variables in the table.

^§^Deployed in support of the operations in Iraq and Afghanistan.

^¶^Indicates reference category.

^#^Self-report of personally witnessing or being exposed to a person's death due to war or disaster, physical abuse, dead and/or decomposing bodies, maimed soldiers or civilians, prisoners of war, or refugees.

**Alcohol misuse is defined as at least 1 positive response to the CAGE questions (i.e., cutting down, annoyance by criticism, guilty feeling, and eye-openers) [32, 33].
